# Immunoglobulin A nephropathy and ischemic heart disease: a nationwide population-based cohort study

**DOI:** 10.1186/s12882-021-02353-7

**Published:** 2021-05-05

**Authors:** Simon Jarrick, Sigrid Lundberg, Johan Sundström, Adina Symreng, Anna Warnqvist, Jonas F. Ludvigsson

**Affiliations:** 1 Department of Pediatrics, Örebro University Hospital, Örebro, Sweden; 2 Faculty of Health and Medicine, Örebro University, Örebro, Sweden; 3 Department of Nephrology, Danderyd Hospital, Stockholm, Sweden; 4 Department of Clinical Sciences, Karolinska Institutet, Danderyd Hospital, Stockholm, Sweden; 5 Department of Medical Sciences, Clinical Epidemiology, Uppsala University, Uppsala, Sweden; 6 The George Institute for Global Health, University of New South Wales, Sydney, Australia; 7 Clinical Epidemiology Division, Department of Medicine, Solna, Karolinska Institutet, Stockholm, Sweden; 8 Division of Biostatistics, Institute of Environmental Medicine, Karolinska Institutet, Stockholm, Sweden; 9 Department of Medical Epidemiology and Biostatistics, Karolinska Institutet, SE-17177 Stockholm, Sweden; 10 Division of Epidemiology and Public Health, School of Medicine, University of Nottingham, Nottingham, UK; 11 Department of Medicine, Columbia University College of Physicians and Surgeons, New York, NY USA

## Abstract

**Background:**

Chronic kidney disease has been linked to cardiovascular disease and specifically ischemic heart disease (IHD), but large-scale population data in patients with immunoglobulin A nephropathy (IgAN) are missing.

**Objective:**

To examine absolute and relative risks for IHD in patients with IgAN.

**Methods:**

Population-based register-based cohort study in Sweden. We identified 3945 patients with biopsy-verified IgAN, and 19,272 age- and sex-matched reference individuals from the general population. To reduce residual confounding from genetic factors and early environmental factors we carried out secondary analyses, where we compared 3039 IgAN patients with 6729 siblings, whereas a spousal analysis consisted of 2377 married couples where one of the spouses had IgAN. Data on IHD and end-stage renal disease (ESRD) were retrieved from the nationwide Patient Register. Cox regression estimated hazard ratios (HRs) adjusted for matching variables, education, country of birth, cancer, diabetes mellitus, and other systemic inflammatory diseases.

**Results:**

During a follow-up of 55,527 person-years (py; mean follow-up 14.1 years), 371 patients (9.4%) with IgAN developed IHD (6.7/1000 py), compared with 1070 (5.6%) in 287,677 py in reference individuals (3.7/1000 py). The corresponding adjusted HR was 1.86 (95%CI = 1.63–2.13), equivalent to one extra case of IHD per 34 IgAN patients followed-up for 10 years. HRs were similar in men and women with IgAN, but higher in the first year after diagnosis and in patients born outside the Nordic countries. Patients with IgAN were at increased risk of IHD also compared to siblings (HR = 2.07; 95%CI = 1.62–2-64) and spouses (HR = 1.91; 95%CI = 1.40–2.61).

**Conclusions:**

In this nationwide population-based study, patients with IgAN were at an 86% increased risk of future IHD.

**Supplementary Information:**

The online version contains supplementary material available at 10.1186/s12882-021-02353-7.

## Introduction

Immunoglobulin A nephropathy (IgAN) is recognized as the most common primary glomerulonephritis worldwide [1, 2], and as such contributes significantly to the global burden of chronic kidney disease (CKD) [2, 3]. As CKD is associated with increased risks of cardiovascular disease and death [4–6], this could be expected also in patients with IgAN. IgAN is also associated with other known risk factors for cardiovascular disease, including hypertension, proteinuria and hyperuricemia [7]. Increases in both systemic and vascular markers of inflammation [8] and in arterial stiffness [9] have been demonstrated even in early IgAN. Nevertheless, the association between cardiovascular disease and IgAN has not been extensively studied, other than in the particular case of end-stage renal disease (ESRD). One study by Myllymäki et al. demonstrated high rates of IHD and cerebrovascular disease in a cohort of 220 patients with biopsy-proven IgAN, although outcomes were not identically defined in exposed and unexposed groups [10].

In a large, nationwide cohort of patients with biopsy-proven IgAN, we recently demonstrated increased overall and cardiovascular mortality, compared with the matched general population [11]. The current study uses the same cohort to examine incident cardiovascular disease in IgAN. The aim of the study was to examine fatal or nonfatal IHD in patients with IgAN compared with a matched reference population, by linking biopsy data to national health registers. Additional comparisons with siblings and spouses allowed us to reduce confounding due to shared risk factors within families and increase our understanding of IgAN and cardiovascular disease even further. We hypothesized that patients are at increased risk of IHD.

## Materials and methods

### Registers

The Swedish personal identity number is a unique 10-digit code assigned to every Swedish resident at birth or immigration. The personal identity number enables large-scale data linkages of multiple national registers [12].

The Swedish Total Population Register [13] contains demographic data on all Swedish residents, including dates of birth, death, immigration and emigration, place of birth and residence, sex, spouse, and educational level. The Total Population Register also includes the Multi-Generation Register, linking all first-degree relatives to all individuals born in Sweden since 1932 who were still alive in 1961.

The Swedish National Patient Register lists hospital stays since 1964, with national coverage since 1987, and hospital outpatient visits since 2001. The register contains information on admission and discharge, main and additional diagnoses, and surgical procedures, with corresponding International Classification of Diseases (ICD) codes and procedure codes. A validation of the Patient Register confirmed a high diagnostic accuracy of 85–95% for most diagnoses, with more severe diagnoses tending to be more accurate [14].

The Swedish Cause of Death Register was started in 1952, with complete coverage since 1961. Registration of the cause of death must be completed within 3 weeks from the death certificate, and is fulfilled in about 99.5% of deceased (the ICD code R99.9 is used for the remaining 0.5%) [15].

The Swedish Prescribed Drug Register contains information about drug expenditures for prescribed drugs in the entire Swedish population, from July 1, 2005, onwards. Hospital-administered drugs and over-the-counter medications are not included. Patient identity data are available for 99.7% of all records [16].

### Definition of IgAN and inclusion of patients

We defined IgAN as having a biopsy record of IgAN (1974–2011) at any of the four pathology departments where all renal biopsy specimens in Sweden are evaluated. In 3 units (Stockholm, Gothenburg, and Linköping), biopsy records where screened for the SnoMed CT (Systematized Nomenclature of Medicine – Clinical Term) codes D67300 (IgAN) and T71000 (kidney) [17]. One unit (Malmö/Lund) did not provide SNOMED codes, and biopsy reports from this region were reviewed manually (see Welander et al. [18] for a detailed description). The resulting cohort of 4125 individuals with a biopsy report of IgAN was validated through manual review of complete patient charts from a random subset of 127 patients, confirming the diagnosis in 95.3% (95% confidence interval (CI): 91.6–98.9%) of the cases [19]. Since we studied first occurrence of IHD as the outcome, we excluded all IgAN patients and all primary and secondary reference individuals with the ICD codes corresponding to IHD before inclusion date (see supplemental eTable S2 for definitions).

### Matched reference individuals (controls)

For each patient with IgAN, the government agency Statistics Sweden (SCB) identified up to five reference individuals from the Total Population Register matched for age, sex, calendar year, and county of residence at the time of renal biopsy in the index patient. Controls with IgAN at inclusion were excluded.

### Secondary comparison groups

To account for residual confounding factors that could not be statistically adjusted for, we used the multigeneration register to identify secondary comparison groups that could be expected to share genetic, environmental and behavioral risk factors with the IgAN patients. Thus, patients with IgAN were compared with siblings and spouses (defined as the first person an IgAN patient was legally married to). IgAN patients without siblings, or who were never married, were excluded from the respective analyses. We also excluded relatives with IgAN at inclusion.

### Follow**-**up

The date of IgAN diagnosis was defined as the date of the first renal biopsy indicating IgAN. Follow-up in IgAN patients began at diagnosis and on the same date in matched reference individuals, siblings and spouses, and ended with studied outcome under examination, death, emigration, or May 312,017, whichever occurred first.

### Outcomes

Our main outcome was the first instance of any IHD, defined as the occurrence of either myocardial infarction or angina pectoris (please see supplemental eTable S2 for corresponding ICD codes) in the patient register, or as the underlying (but not as of the contributory) cause in the Cause of Death Register. We also studied myocardial infarction separately.

### Covariates

We collected information on relevant demographic and clinical factors present at inclusion, which could influence cardiovascular risk as possible confounders. Data on sex, country of birth, and educational level were retrieved from the Total Population Register. Data on relevant comorbidity (diabetes, cancer, systemic inflammatory diseases other than IgAN and cardiovascular disease other than IHD and hypertension) were extracted from the Patient Register. Heredity for IgAN was defined as having ≥1 first-degree relative with a biopsy report of IgAN before study entry of the index individual. First-degree relative were identified through the multigeneration register and did not include spouses.

ESRD was defined as the first occurrence in the patient register of any ICD code for CKD stage 5, or any ICD code or procedure code associated with dialysis or kidney transplantation (supplemental eTable S3). To increase specificity, three or more records of an ESRD diagnosis were required, with at least 4 months between the first and the last diagnosis.

As hypertension is commonly caused by chronic kidney disease including IgAN, and a known risk factor for IHD, it was considered as a mediator rather than a confounder and not adjusted for in the models.

### Statistical analysis

We used Cox regression with internal stratification (each IgAN individual was compared with his or her matched reference individuals) to calculate hazard ratios (HRs) for any IHD (our main outcome) as well as myocardial infarction. Since proportionality over time was not verified statistically, HRs were considered as estimates of the geometric mean of the true, changing HRs [20], but we also presented HRs in the first year of follow-up.

Event-free survival was presented graphically using Cox adjusted survival diagrams.

All analyses were adjusted for educational level (≤ 9, 9–12, and ≥ 13 years), country of birth (Nordic, including Sweden, vs. non-Nordic countries), and the presence at baseline of cardiovascular disease, cancer, diabetes mellitus, and other systemic inflammatory diseases (supplementary eTable S1 for definitions). Comparisons with general population reference individuals were also adjusted for heredity for IgAN and ESRD. As sibling and spousal analyses could not be matched for sex and age, these analyses were instead adjusted for in the Cox model. Throughout the study, we exclusively report adjusted HRs. We also calculated IHD- and disease-specific incidence rates per 1000 person-years (py) in IgAN patients and reference individuals, and present absolute excess rates (unadjusted).

We used Stata Statistical Software, release 15 (StataCorp LP, College Station, TX) for the statistical calculations. *P* values < 0.05 were considered statistically significant.

### Ethics

The study was approved by the Stockholm Ethics Review Board (January 22, 2014; Dnr 2013/2095–31/2). Because this was a strictly register-based study, informed consent was waivered by the Board [21]. The current study was carried out in accordance with relevant guidelines and regulations..

## Results

### Demographics and background variables

We identified 4125 individuals with IgAN. After exclusion of 180 individuals with IHD before inclusion date, 3945 were included in the final analyses (Table 1). Extrarenal manifestations of IgA vasculitis (Henoch-Schönlein purpura) were present in 170 (4.3%). Those 3945 patients were compared to 19,272 matched reference individuals (main analysis). The sibling analysis compared 3039 IgAN patients with 6729 siblings, whereas the spousal analysis consisted of 2377 married couples.

**Table 1:** Descriptive baseline characteristics of patients with IgAN compared to matched general population reference individuals, siblings and spouses

	Main analysis		Sibling analysis		Spousal analysis	
	IgAN	General population reference	IgAN	Siblings	IgAN	Spouses
	*n* (%)	*n* (%)	*n* (%)	*n* (%)	*n* (%)	*n* (%)
Total	3945	19,272	3039	6729	2377	2377
Person-years (py)	55,527	287,677	44,796	105,096	35,484 (0)	38,893 (0)
**Sex**						
Female	1172 (29.7%)	5780 (30.0%)	869 (28.6%)	3307 (49.1%)	690 (29.0%)	1710 (71.9%)
Male	2773 (70.3%)	13,492 70.0%)	2170 (71.4%)	3422 (50.9%)	1687 (71.0%)	667 (28.1%)
**Age**						
Age, mean (SD)	39.4 (16.5)	38.9 (16.2)	35.9 (14.9)	35.3 (16.6)	43.5 (14.3)	42.9 (14.6)
Age, median (IQR)	38.9 (26.5, 51.3)	38.3 (26.2, 50.5)	35.3 (23.9, 47)	35.7 (23, 47.9)	43.1 (32.7, 53.9)	42.8 (32.3, 53.2)
≤ 17 years	380 (9.6%)	1894 (9.8%)	361 (11.9%)	1093 (16.2%)	64 (2.7%)	91 (3.8%)
18–39 years	1706 (43.2%)	8541 (44.3%)	1485 (48.9%)	2872 (42.7%)	943 (39.7%)	947 (39.8%)
40–59 years	1390 (35.2%)	6778 (35.2%)	1015 (33.4%)	2312 (34.4%)	1046 (44.0%)	1036 (43.6%)
≥ 60 years	469 (11.9%)	2059 (10.7%)	178 (5.9%)	452 (6.7%)	324 (13.6%)	296 (12.5%)
Unknown					0 (0.0%)	7 (0.3%)
**Year of inclusion**						
Entry year, median (IQR)	2001 (1994, 2007)	2001 (1994, 2007)	2002 (1994, 2008)	2001 (1994, 2007)	2000 (1993, 2006)	2000 (1993, 2006)
1974–1988	438 (11.1%)	2168 (11.2%)	317 (10.4%)	728 (10.8%)	326 (13.7%)	326 (13.7%)
1989–2001	1565 (39.7%)	7662 (39.8%)	1187 (39.1%)	2670 (39.7%)	1024 (43.1%)	1024 (43.1%)
2002–2015	1942 (49.2%)	9442 (49.0%)	1535 (50.5%)	3331 (49.5%)	1027 (43.2%)	1027 (43.2%)
**Follow up time**						
Mean (SD)	14.1 (8.47)	14.9 (8.52)	14.7 (8.49)	15.6 (8.51)	14.9 (8.76)	16.4 (8.67)
Median (IQR)	12.7 (7.45, 19.6)	13.7 (8.11, 20.7)	13.5 (7.99, 20.4)	14.5 (8.61, 21.5)	13.8 (8.01, 20.7)	15.5 (9.3, 22.5)
0–1 years	79 (2.0%)	194 (1.0%)	35 (1.2%)	41 (0.6%)	56 (2.4%)	20 (0.8%)
1–4 years	464 (11.8%)	2001 (10.4%)	314 (10.3%)	569 (8.5%)	248 (10.4%)	176 (7.4%)
≥ 5 years	3402 (86.2%)	17,077 88.6%)	2690 (88.5%)	6119 (90.9%)	2073 (87.2%)	2181 (91.8%)
**Reason for end of follow up**						
May 31, 2017	3022 (76.6%)	16,000 83.0%)	2524 (83.1%)	6020 (89.5%)	1759 (74.0%)	2021 (85.0%)
Emigration	105 (2.7%)	727 (3.8%)	80 (2.6%)	160 (2.4%)	48 (2.0%)	45 (1.9%)
IHD (death or diagnosis)	371 (9.4%)	1070 (5.6%)	206 (6.8%)	242 (3.6%)	290 (12.2%)	126 (5.3%)
Death from other cause	447 (11.3%)	1475 (7.7%)	229 (7.5%)	307 (4.6%)	280 (11.8%)	185 (7.8%)
**Country of birth**						
Nordic	3602 (91.3%)	17,322 89.9%)	2971 (97.8%)	6568 (97.6%)	2128 (89.5%)	2085 (87.7%)
Non-Nordic	342 (8.7%)	1948 (10.1%)	68 (2.2%)	161 (2.4%)	249 (10.5%)	229 (9.6%)
Unknown	1 (< 1%)	2 (< 1%)	0 (0.0%)	0 (0.0%)	0 (0.0%)	63 (2.7%)
**Level of education**						
Compulsory school (0–9 years)	817 (20.7%)	3852 (20.0%)	506 (16.7%)	1195 (17.8%)	527 (22.2%)	435 (18.3%)
Upper sec. School (10–12 years)	1759 (44.6%)	8690 (45.1%)	1415 (46.6%)	3205 (47.6%)	1007 (42.4%)	1044 (43.9%)
University (> 12 years)	1276 (32.3%)	6279 (32.6%)	1066 (35.1%)	2090 (31.1%)	816 (34.3%)	817 (34.4%)
Unknown	93 (2.4%)	451 (2.3%)	52 (1.7%)	239 (3.6%)	27 (1.1%)	81 (3.4%)
**Comorbitity**^**a**^						
HSP/IgAV^b^	236 (6.0%)	30 (0.2%)	203 (6.7%)	22 (0.3%)	94 (4.0%)	5 (0.2%)
Diabetes, type 1 or 2	170 (4.3%)	309 (1.6%)	97 (3.2%)	107 (1.6%)	101 (4.2%)	45 (1.9%)
Other systemic inflammatory disease	313 (7.9%)	459 (2.4%)	224 (7.4%)	207 (3.1%)	193 (8.1%)	77 (3.2%)
Hypertension	1241 (31.5%)	344 (1.8%)	891 (29.3%)	139 (2.1%)	809 (34.0%)	51 (2.1%)
Heredity for IgAN	38 (1.0%)	28 (0.1%)				
Heredity for ESRD	84 (2.1%)	139 (0.7%)				

Continuous parameters are presented as mean values with standard deviations. Categorical values are presented as numbers and percentages. For all definitions, see main text or Additional file 1

^a^Before study inclusion date (biopsy date in IgAN patients and corresponding date in reference individuals)

^b^*HSP/IgAV* Henoch-Schönlein’s purpura, or systemic IgA vasculitis

In the main analysis, the median age at inclusion in both groups was 39 years. The male to female ratio among IgAN patients was 2.4:1. The mean (standard deviation) follow-up time was 14.1 (8.5) years for IgAN patients versus 14.9 (8.5) years for reference individuals, with similar follow-up in sibling and spousal analyses. Other demographic characteristics were similar between groups. Among IgAN patients, 1241 (31.5%) had a diagnosis of hypertension, compared to 344 (1.8%) among matched reference individuals. Diabetes and concomitant systemic inflammatory disease were more common in IgAN patients compared to all comparison groups.

Hypertension was present in 31.5% of the IgAN patients and 1.8% of the reference individuals at inclusion, and 50.1% of IgAN patients had a hypertension diagnosis any time during follow-up. Among 1343 IgAN patients included after July 12,005 when we had access to data on medication, glucocorticoids were prescribed to 784 (58.4%), and other immunosuppressive drugs to 345 (25.7%) patients during follow-up, and for 178 (13.3%) and 93 (6.9%) patients during the first year after biopsy. Renin-angiotensin-aldosterone system inhibitors (RAASi) were used in 1172 (87.3%) and other antihypertensive drugs in 841 (62.3%) IgAN patients, and in 516 (38.4%) and 354 (26.4%) respectively during the first year. Statins were prescribed to 578 (43.0%) patients during follow-up, and to 162 (12.1%) in the first year (supplemental eTable S4).

### Main outcome: any ischemic heart disease (IHD)

**Fig. 1:**
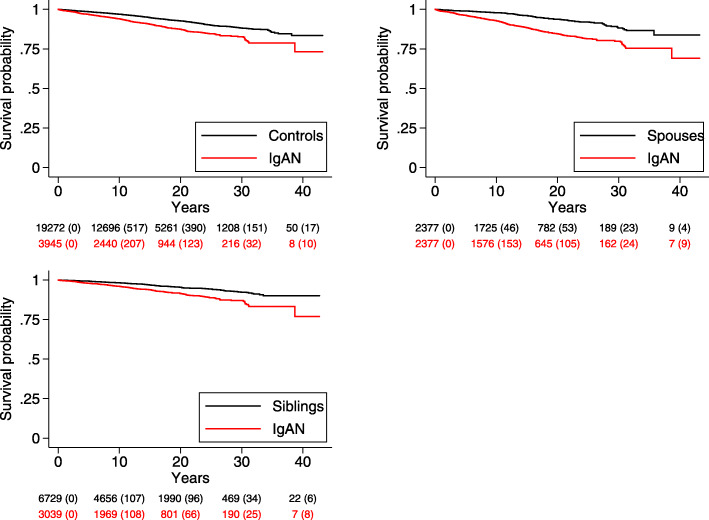
Event-free survival in patients with IgA nephropathy, compared with matched reference individuals, siblings and spouses, presented as Kaplan-Meier diagrams, with ischemic heart disease as outcome

**Fig. 2:**
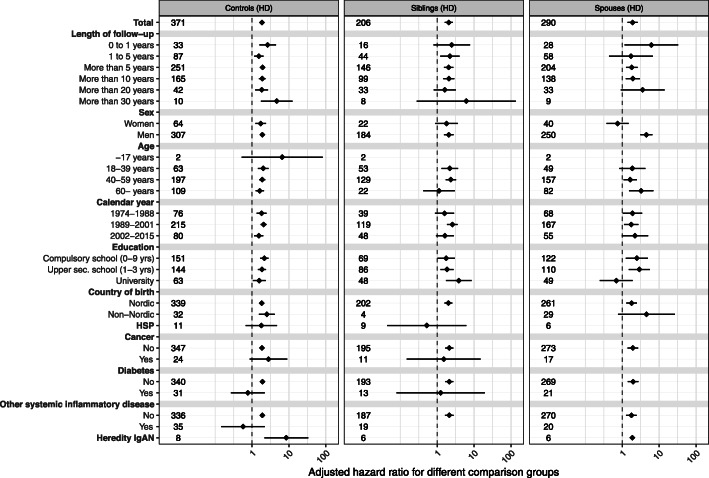
Forrest plots presenting adjusted hazard ratios for ischemic heart disease in IgA nephropathy compared to general population controls, siblings and spouses. Legend: HSP, Henoch-Schönlein Purpura

During a follow-up of 55,527 person-years (py), 371 patients (9.4%) with IgAN had at least one record of any IHD (6.7 per 1000 py), compared with 1070 (5.6%) during 287,677 py in reference individuals (3.7 per 1000 py) (Figs. 1 and 2, supplemental eTable S5). The unadjusted absolute excess rate was 3.0 per 1000 py. The corresponding adjusted HR was 1.86 (95%CI = 1.63–2.13), equivalent to one extra case of IHD per 34 IgAN patients followed-up for 10 years. The HRs were similar across subgroups. The rate of IHD was higher in the first year after inclusion (8.5 per 1000 py; HR = 2.63 (95%CI = 1.57–4.41)), and increased both among Nordic (6.58 per 1000 py; HR = 1.83 (95%CI = 1.59–2.1)) and non-Nordic individuals (7.97 per 1000 py; HR = 2.52 (95%CI = 1.53–4.14)). The HR for IHD did not differ significantly between women (1.72; 95%CI = 1.24–2.37) and men (1.90; 95%CI = 1.64–2.21). For all other strata, HRs were of the same magnitude compared to the general population reference individuals. Risk estimates were generally similar when IgAN patients were compared to their siblings (HR = 2.07; 95%CI = 1.62–2-64) (supplemental eTable S6) and spouses (HR = 1.91; 95%CI = 1.40–2.61), with the exception of a significantly higher HR for IHD in men than in women in spousal analysis (supplemental eTable S7).

### Secondary outcome: myocardial infarction (MI)

Restricting the outcome to myocardial infarction (MI), there were 216 events (5.5%) during 56,865 py in IgA patients, versus 662 events (3.4%) during 291,433 py in reference individuals, with an unadjusted absolute excess rate of 1.5 per 1000 py (one case in 65 individuals followed for 10 years), and a corresponding adjusted HR (aHR) of 1.83 (95%CI = 1.54–2.18) (supplemental eTable S8). Non-Nordic origin was associated with a higher aHR (3.67; 95%CI = 1.37–9.84).

## Discussion

In the current nation-wide, population-based study including more than 3900 patients diagnosed with IgAN in Sweden over a study period of 37 years, we found an 86% increased risk of IHD, equivalent to one extra case of IHD per 34 IgAN patients followed-up for 10 years. The IHD excess risk was present across a range of IgAN sub-populations and compared to siblings and spouses. Similar HRs were seen for myocardial infarction.

Previous studies have shown vascular changes with increased arterial stiffness in IgAN patients even at early stages [9, 22]. Consistently, we found hypertension in almost one third of IgAN patients already at the time of biopsy, compared to less than 2 % in a matched reference population, and half of the patients had hypertension during follow up (according to ICD code; 87% were prescribed RAAS inhibitors, which might have been intended also for treatment of proteinuria, and 62% other antihypertensive drugs). There is also robust evidence that CKD patients experience a risk increase for cardiovascular events and death with increasing CKD stage, long before they reach ESRD [4–6, 23]. Indeed, one Finnish study found tripled odds for cardiovascular disease in IgAN patients compared with an age- and sex-matched reference population derived from national health survey data and doubled odds when only IgAN patients with stable non-progressive kidney disease were included [10]. The deviation from our results might in part be due to a different patient selection (we used national biopsy data and did not rely on tertiary center data), our use of biopsy records to define IgAN (positive predictive value = 95% [19]), and that the Finnish study derived their reference population from survey interviews, while we relied on national health registers with physician-reported data.

After adjusting for potential confounders including prevalent comorbidity, we found similar HRs in both men (1.72) and women (1.90). We also noted a strong association between IgAN and IHD in the first year after IgAN. This may reflect a more intense clinical work-up for symptoms suggestive of IHD when the IgAN patients have frequent healthcare visits. We chose to examine IHD-naïve IgAN patients and hence excluded individuals with a prior IHD diagnosis. We did so to be able to estimate the future risk of IHD in IgAN, a topic of interest to many patients.

### Strengths and limitations

We followed more than 3900 IgAN patients and 19,200 reference individuals for a mean follow up time of > 14 years. Through linkage to the personal identity number we virtually eliminated loss of follow-up using nationwide registers on emigration status, and death. The number of IHD cases in IgAN (*n* = 371) to our knowledge exceeds the total number reported in the literature up to date. This allowed us to calculate both relative and absolute risks with substantial precision. We were able to demonstrate a clearly increased risk (HR = 1.86), but also to rule out huge excess risks (the upper 95%CI in our study signals that a more than 2.2-fold risk increase is unlikely). Our absolute risk estimates will help physicians convey an understandable message to IgAN patients (one extra case of IHD in 34 IgAN patients followed-up for 10 years). The nationwide, population-based design should minimize selection bias. The Swedish national health registers are well validated and of high quality. Through the Patient Register (positive predictive value for most disorders have been estimated at 85–95%) [14], we retrieved information on important potential confounders such as diabetes, other inflammatory conditions and cancer. Furthermore, our data were adjusted for education and country of birth. Additionally, the large number of study participants enabled us to carry out stratified analyses which will allow clinicians to optimize information (same HR in women and men but higher HRs in the first year after diagnosis). The inclusion of a reference population matched for sex and calendar year enabled us to perform Cox regression with internal stratification. We compared IgAN patients not only with the general population, but also with their siblings and spouses. Very similar HRs in these secondary comparisons indicate that the increase in IHD is related to IgAN, and not to shared genetic and early environmental factors. Finally, our exposure was confirmed through biopsy. In an earlier validation study, 121/127 (95%) randomly selected IgAN patents from our cohort had IgAN according to patient chart records. IgA deposits were reported in 97% of the biopsy records (*n* = 123), mesangial hypercellularity in 76% (*n* = 96), C3 deposits in 89% (*n* = 113).

We also acknowledge some limitations. We did not have information on cardiovascular risk factors such as glomerular filtration rate, proteinuria or precise blood pressure levels, but it can be argued that these intermediates should not be adjusted for, as these factors are to be considered mediators rather than confounders. Still, stratification on such markers of disease severity could have deepened understanding of risk estimates. We also lack information on smoking. As previous studies have found similar smoking patterns in IgAN patients and the general population [24], smoking habits are unlikely to have biased our results. Except for IgAN related to Henoch Schönlein purpura/IgA vasculitis, our data did not let us distinguish primary IgA from secondary forms.

In conclusion, this nationwide population-based study found an 86% increased risk of future IHD in patients with IgAN.

## Supplementary Information

**Additional file 1: Table S1. **Internationalclassification of disease (ICD) codes for renal disease, cardiovasculardisease, diabetes, cancer, Henoch-Schönlein purpura and other systemicinflammatory diseases. **Table S2.** International classification of disease (ICD) codes for ischemic heart disease. **Table S3.** Definitionof renal endpoints. **Table S4.** AnatomicalTherapeutical Chemical (ATC) codes for medications, used for matching to theSwedish Prescribed Drugs register. **Table S5.** Adjustedhazardratios (HRs) for *ischemic heart disease*compared with the *general referencepopulation* (corresponding to column 1 in Fig. 2). **Table S6.** Adjustedhazardratios (HRs) for *ischemic heart disease*compared with *siblings* (correspondingto column 2 in Fig. 2). **Table S7.** Adjustedhazardratios (HRs) for *ischemic heart disease*compared with *spouses* (correspondingto column 3 in Fig. 2). **Table S8.** Adjustedhazard ratios (HRs) for *acute myocardialinfarction* compared with the *generalreference population.*

## Data Availability

The data that support the findings of this study are available from the National Board of Health and Welfare, the government agency Statistics Sweden, and the Pathology departments delivering IgAN data, but restrictions apply to the availability of these data, which were used under license for the current study, and so are not publicly available. Data are however available from the authors upon reasonable request and with permission of the National Board of Health and Welfare, the government agency, Statistics Sweden, and the Pathology departments delivering IgAN data.

## Abbreviations

CKD: Chronic kidney disease
ERSD: End-stage Renal disease
IgAN: Immunoglobulin A nephropathy
IHD: Ischemic heart disease
