# Supplementary material for: Immunoglobulin A nephropathy and ischemic heart disease: a nationwide population-based cohort study
Source: BMC Nephrol. 2021 May 5;22:165. doi: 10.1186/s12882-021-02353-7 (PMC8101187; doi:10.1186/s12882-021-02353-7)
Supplement: Supplementary file 1 — Additional file 1: Table S1. Internationalclassification of disease (ICD) codes for renal disease, cardiovasculardisease, diabetes, cancer, Henoch-Schönlein purpura and other systemicinflammatory diseases. Table S2. International classification of disease (ICD) codes for ischemic heart disease. Table S3. Definitionof renal endpoints. Table S4. AnatomicalTherapeutical Chemical (ATC) codes for medications, used for matching to theSwedish Prescribed Drugs register. Table S5. Adjustedhazardratios (HRs) for ischemic heart diseasecompared with the general referencepopulation (corresponding to column 1 in Fig. 2). Table S6. Adjustedhazardratios (HRs) for ischemic heart diseasecompared with siblings (correspondingto column 2 in Fig. 2). Table S7. Adjustedhazardratios (HRs) for ischemic heart diseasecompared with spouses (correspondingto column 3 in Fig. 2). Table S8. Adjustedhazard ratios (HRs) for acute myocardialinfarction compared with the generalreference population. [file 12882_2021_2353_MOESM1_ESM.doc]

**SUPPLEMENTARY APPENDIX**

Immunoglobulin A nephropathy and ischemic heart disease: A nationwide population-based cohort study

Simon Jarrick, MD, Sigrid Lundberg, MD, PhD, Johan Sundström, MD, PhD, Adina Symreng, MD, PhD, Anna Warnqvist, MSc, Jonas F. Ludvigsson, MD, PhD

Table of Contents

| Page 2 | **Table S1.** International classification of disease (ICD) codes for renal disease, cardiovascular disease, diabetes, cancer, Henoch-Schönlein purpura and other systemic inflammatory diseases |
| --- | --- |
|  | **Table S2.** International classification of disease (ICD) codes for ischemic heart disease.  **Table S3.** Definition of renal endpoints.  **Table S4.** Anatomical Therapeutical Chemical (ATC) codes for medications, used for matching to the Swedish Prescribed Drugs register. |
| Page 4 | **Table S5.**  Adjusted hazard ratios (HRs) for *ischemic heart disease* compared with the *general reference population* (corresponding to column 1 in Fig. 2). |
| Page 5 | **Table S6.**  Adjusted hazard ratios (HRs) for *ischemic heart disease* compared with *siblings* (corresponding to column 2 in Fig. 2). |
| Page 6 | **Table S7.**  Adjusted hazard ratios (HRs) for *ischemic heart disease* compared with *spouses* (corresponding to column 3 in Fig. 2) |
| Page 7 | **Table S8.** Adjusted hazard ratios (HRs) for *acute myocardial infarction* compared with the *general reference population.* |
| Page 8 | **Strobe statement** |
|  |  |
|  |  |
|  |  |
|  |  |
|  |  |
|  |  |
|  |  |
|  |  |

**eTable S1.** International classification of disease (ICD) codes for renal disease, cardiovascular disease, diabetes, cancer, autoimmune disease, Henoch-Schönlein purpura (HSP) and other systemic inflammatory diseases.

| **Diagnostic group** | **ICD-8** | **ICD-9** | **ICD-10** |
| --- | --- | --- | --- |
| **Heredity for renal disease at study entry (adjusted for)** | | | |
| End-stage renal disease | see eTable S2 | | |
| IgA Nephropathy | defined according to renal biopsy records | | |
| **IgA Nephropathy phenotype** | | | |
| Concurrent extra-renal IgA vasculitis (Henoch-Schönlein purpura) before or <1 year after IgAN diagnosis | 287,00 | 278A | D69.0 |
| **Comorbidity at study entry (adjusting for 1-4)** | | | |
| 1. Cardiovascular disease/death | 390-398; 420-458 | 390-398, 415-417, 420-459 | I00-I02; I26-99 |
| 2. Cancer | 140-209 | 140-208 | C00-D48 |
| 3. Diabetes mellitus (type 1 or 2) | 250 | 250 | E10-14 |
| 4. Other systemic inflammatory diseases (any of those below but other than diabetes) |  |  |  |
| *-Psoriasis* | 696 (969,30 excluded) | 696A-C + E-W | L40 |
| *-SLE* | 734,1 | 710A | M32.1; M32.8; M32.9 |
| *-Rheumatoid arthritis* | 712,3; 714,93 | 714 | M05; M06; M08; M09; M12.3 |
| *-Crohn’s disease* | 572,00; 572,09; 563,00 | 555 | K50 |
| *-Ulcerative colitis* | 572,20; 572,21; 563,10 | 556 | K51 |
| *-Thyroiditis* | 245 | 245A-X | E06 |
| *-Hyperthyroidism* | 242 | 242 | E05 |
| *-Sarcoidosis* | 135 | 135 | D86 |
| *-Primary biliary cirrhosis* |  | 571G | K74.3 |
| *-ANCA vasculitis and other vasculitis* | 446, | 446 | M31 |
| *-Celiac disease* | 269,0 | 579A | K90.0 |
| *-Pelvospondylitis* |  |  | M45.9 |
| *-Autoimmune hepatitis* | 573,08; 573,09 | 573D | K75.4; K75.5; K75.9 |
| *-Primary sclerosing cholangitis* |  |  | K83.0A |
| 5. Hypertension | 400-404 | 400-405 | I10-15 |

**eTable S2.** International classification of disease (ICD) codes for ischemic heart disease.

| **Outcomes^a^** | **ICD8** | **ICD9** | **ICD10** |
| --- | --- | --- | --- |
| Myocardial infarction^b, c^ | 410 | 410 | I21, I22 |
| Angina pectoris^b, c^ | 411 | 411B, 413, 414A | I20 |

^a^ According to *any* diagnosis in the Swedish Patient Register or *underlying* cause but not contributory cause in the Causes of Death Register.

^b^ Myocardial infarction and Angina Pectoris, together constituted the main outcome measure Ischemic heart disease.

^c^ Separate risk estimates are calculated for these diagnoses.

**eTable S3.** International classification of disease (ICD) codes for renal endpoints using hospital-based in- or outpatient diagnosis.

| **Diagnostic group** | **ICD-8** | **ICD-9** | **ICD-10** | **Procedure codes** |
| --- | --- | --- | --- | --- |
| Medical diagnosis of end-stage renal disease (ESRD)^a^ | - | 585 | N18.0; N18.5 |  |
| Renal dialysis^b^ | Y29,01 | V45B; V56 | Z49; Z99.2 | 9200; V9200; 9212; V9212; 9314; V9531; DR012; DR013; DR016; DR024; QF006 |
|  |  |  |  | 9211; V9211; 9213; V9213; V9532; DR015; DR023; DR055; DV056 |
|  |  |  |  | 9219; V9219; 9223; V9223; DR017; DR020; DR055; DR056 |
| Renal transplantation |  | V42A | Z94.0 | 6070; KAS10; KAS20 |

^a^ In the Patient Register or the Cause of Death Register (underlying or contributory cause).

^b^ Regarded as ESRD if *occurring ≥3 times in a patient with ≥4 months between the first and last dialysis*.

**Table S4.** Anatomical Therapeutical Chemical (ATC) codes for medications, used for matching to the Swedish Prescribed Drugs register.

| **Medication class** | **ATC code** |
| --- | --- |
| Glucocorticoids for systemic use | H02AB, except H02AB08 (triamcinolone)  H02B |
| Immunosuppressive drugs, other | Cyclophosphamide (L01AA01);  Rituximab (L01XC02)  Mycophenolic acid (L04AA06)  Cyclosporine (L04AD01  Tacrolimus (L04AD02)  Azathioprine (L04AX01), |
| Inhibitors of the renin-angiotensin-aldosterone system (RAAS inhibitors). | C09 |
| Anti-hypertensive drugs, other | C02, C03 C07 and C08 |
| Statins | C10AA |

**eTable S5.** Risk of *ischemic heart disease (composite outcome)^a^* in patients with IgA nephropathy (IgAN) diagnosed in Sweden from 1974-2011 compared with the *general reference population* (corresponds to Fig. 2, column 1).

| **Variable** | **IgAN: Events/py** | **Ref: Events/py** | **Events/1000 py: IgAN vs. Ref** | **Adjusted HR (95% CI)** |
| --- | --- | --- | --- | --- |
| Total | 371/55527 | 1070/287677 | 6.7 vs. 3.7 | 1.86 (1.63, 2.13) |
| **Length of follow-up** | | | | |
| 0 to 1 years | 33/3897 | 49/19166 | 8.5 vs. 2.6 | 2.63 (1.57, 4.41) |
| 1 to 5 years | 87/18554 | 224/92032 | 4.7 vs. 2.4 | 1.53 (1.14, 2.04) |
| > 5 years^b^ | 251/53942 | 797/280895 | 4.7 vs. 2.8 | 1.92 (1.63, 2.25) |
| > 10 years^b^ | 165/46648 | 557/247500 | 3.5 vs. 2.3 | 1.89 (1.55, 2.31) |
| > 20 years^b^ | 42/24744 | 167/138354 | 1.7 vs. 1.2 | 1.82 (1.22, 2.72) |
| > 30 years^b^ | 10/7143 | 16/40094 | 1.4 vs. 0.4 | 4.65 (1.73, 12.5) |
| **Sex** | | | | |
| Women | 64/17115 | 199/87340 | 3.7 vs. 2.3 | 1.72 (1.24, 2.37) |
| Men | 307/38412 | 871/200337 | 8.0 vs. 4.3 | 1.90 (1.64, 2.21) |
| **Age** | | | | |
| ≤ 17 years | 2/5905 | 3/29913 | 0.34 vs. 0.1 | 6.56 (0.522, 82.4) |
| 18-39 years | 63/28103 | 147/141317 | 2.2 vs. 1 | 2.01 (1.45, 2.78) |
| 40-59 years | 197/17826 | 570/95338 | 11 vs. 6 | 1.88 (1.57, 2.27) |
| ≥ 60 years | 109/3694 | 350/21109 | 30 vs. 17 | 1.62 (1.25, 2.11) |
| **Calendar year of inclusion** | | | | |
| 1974-1988 | 76/11404 | 270/59934 | 6.7 vs. 4.5 | 1.81 (1.34, 2.46) |
| 1989-2001 | 215/27414 | 569/143982 | 7.8 vs. 4 | 2.05 (1.72, 2.46) |
| 2002-2015 | 80/16710 | 231/83761 | 4.8 vs. 2.8 | 1.52 (1.14, 2.01) |
| **Education** | | | | |
| Compulsory school (0-9 yrs) | 151/10641 | 385/58970 | 14 vs. 6.5 | 2.15 (1.67, 2.78) |
| Upper sec. school (1-3 yrs) | 144/25758 | 405/131213 | 5.6 vs. 3.1 | 1.86 (1.45, 2.38) |
| University | 63/18538 | 223/92367 | 3.4 vs. 2.4 | 1.57 (1.05, 2.35) |
| **Country of birth** | | | | |
| Nordic | 339/51507 | 998/266094 | 6.6 vs. 3.8 | 1.83 (1.59, 2.1) |
| Non-Nordic | 32/4015 | 72/21479 | 8.0 vs. 3.3 | 2.52 (1.53, 4.14) |
| **Henoch-Schönlein purpura (systemic IgA vasculitis)^c^** | | | | |
| Yes | 11/2678 | 26/13440 | 4.1 vs. 1.9 | 1.78 (0.675, 4.71) |
| **Cancer^d^** | | | | |
| No | 347/53132 | 1000/274933 | 6.5 vs. 3.6 | 1.86 (1.62, 2.14) |
| Yes | 24/2395 | 70/12745 | 10 vs. 5.5 | 2.77 (0.858, 8.95) |
| **Diabetes^d^** | | | | |
| No | 340/54075 | 1000/278986 | 6.3 vs. 3.6 | 1.91 (1.66, 2.2) |
| Yes | 31/1452 | 70/8691 | 21 vs. 8.1 | 0.77 (0.269, 2.2) |
| **Other systemic inflammatory disease^d^** | | | | |
| No | 336/52296 | 965/269644 | 6.4 vs. 3.6 | 1.90 (1.65, 2.19) |
| Yes | 35/3231 | 105/18033 | 11 vs. 5.8 | 0.569 (0.147, 2.21) |
| **Cardiovascolar disease (other than hypertension and IHD)^d^** | | | | |
| No | 286/50018 | 823/256744 | 5.7 vs. 3.2 | 1.95 (1.67, 2.27) |
| Yes | 85/5509 | 247/30933 | 15 vs. 8.0 | 1.11 (0.72, 1.72) |
| **First-degree relative with IgAN** | | | | |
| Yes | 8/4497 | 10/2551 | 18 vs. 3.9 | 8.46 (2.15, 33.3) |

^a^ For definition of ischeamic heart disease, see eTable S3

^b^ Not mutually exclusive. For instance a patient with a follow-up of 11 years would be included in both the >5-year- and the >10-year-categories.

^c^ Before or within 1 year after first renal biopsy indicating IgAN.

^d^ Before study entry (date of IgAN and corresponding date in reference individuals). For definitions, see table S1

^e^ Adjusted HR not possible to estimate due to lack of variation within strata.

py = person-years, HR = hazard ratio.

**eTable S6**: Risk of *ischemic heart disease (composite outcome)^a^* in patients with IgA nephropathy (IgAN) diagnosed in Sweden in 1974-2011, compared with *siblings* (corresponds to Fig. 2, column 2).

| **Variable** | **IgAN: Events/py** | **Ref: Events/py** | **Events/1000 py: IgAN vs. Ref** | **HR (95% CI)** |
| --- | --- | --- | --- | --- |
| Total | 206/44796 | 242/105096 | 4.6 vs. 2.3 | 2.07 (1.62, 2.64) |
| **Length of follow-up** | | | | |
| 0 to 1 years | 16/3017 | 11/6704 | 5.3 vs. 1.6 | 2.48 (0.794, 7.75) |
| 1 to 5 years | 44/14518 | 37/32564 | 3.0 vs. 1.1 | 2.22 (1.21, 4.06) |
| > 5 years^a^ | 146/43709 | 194/103090 | 3.3 vs. 1.9 | 2.04 (1.53, 2.72) |
| > 10 years^a^ | 99/38216 | 136/91887 | 2.6 vs. 1.5 | 2.07 (1.47, 2.92) |
| > 20 years^a^ | 33/21042 | 40/52624 | 1.6 vs. 0.76 | 1.61 (0.814, 3.18) |
| > 30 years^a^ | 8/6290 | 6/15614 | 1.3 vs. 0.38 | 6.14 (0.28, 135) |
| **Sex** | | | | |
| Women | 22/13356 | 59/30132 | 1.6 vs. 2 | 1.79 (0.884, 3.65) |
| Men | 184/31440 | 183/74964 | 5.8 vs. 2.4 | 2.06 (1.53, 2.78) |
| **Age** | | | | |
| ≤ 17 years | 2/5662 | 0/12304 | 0.35 vs. 0 | **e** |
| 18-39 years | 53/24608 | 69/55327 | 2.2 vs. 1.3 | 2.19 (1.32, 3.65) |
| 40-59 years | 129/13185 | 145/34017 | 9.8 vs. 4.3 | 2.35 (1.71, 3.22) |
| ≥ 60 years | 22/1341 | 28/3447 | 16 vs. 8.1 | 1.12 (0.417, 3.02) |
| **Calendar year of inclusion** | | | | |
| 1974-1988 | 39/9066 | 54/21706 | 4.3 vs. 2.5 | 1.59 (0.876, 2.87) |
| 1989-2001 | 119/22280 | 137/52886 | 5.3 vs. 2.6 | 2.58 (1.86, 3.58) |
| 2002-2015 | 48/13450 | 51/30504 | 3.6 vs. 1.7 | 1.61 (0.925, 2.81) |
| **Education** | | | | |
| Compulsory school (0-9 yrs) | 69/7105 | 75/18861 | 9.7 vs. 4 | 1.75 (1.03, 2.98) |
| Upper sec. school (1-3 yrs) | 86/21570 | 113/52053 | 4.0 vs. 2.2 | 1.85 (1.23, 2.80) |
| University | 48/15745 | 53/32773 | 3.0 vs. 1.6 | 3.86 (1.74, 8.60) |
| **Country of birth** | | | | |
| Nordic | 202/43944 | 240/102366 | 4.6 vs. 2.3 | 2.02 (1.57, 2.58) |
| Non-Nordic | 4/852 | 2/2729 | 4.7 vs. 0.73 | **e** |
| **Henoch-Schönlein purpura (systemic IgA vasculitis)^c^** | | | | |
| Yes | 9/2363 | 9/5104 | 3.8 vs. 1.8 | 0.522 (0.044, 6.12) |
| **Cancer^d^** | | | | |
| No | 195/42962 | 228/100821 | 4.5 vs. 2.3 | 2.12 (1.65, 2.73) |
| Yes | 11/1834 | 14/4275 | 6.0 vs. 3.3 | 1.50 (0.15, 15.1) |
| **Diabetes^d^** | | | | |
| No | 193/43852 | 235/102750 | 4.4 vs. 2.3 | 2.14 (1.66, 2.75) |
| Yes | 13/944 | 7/2345 | 14 vs. 3.0 | 1.24 (0.079, 19.6) |
| **Other systemic inflammatory disease^d^** | | | | |
| No | 187/42398 | 221/99141 | 4.4 vs. 2.2 | 2.13 (1.64, 2.76) |
| Yes | 19/2398 | 21/5955 | 7.9 vs. 3.5 | **e** |
| **Cardiovascular disease (other than hypertension and IHD)^d^** | | | | |
| No | 165/41105 | 201/95131 | 4.0 vs. 2.1 | 2.30 (1.75, 3.03) |
| Yes | 41/3690 | 41/9965 | 11 vs. 4.1 | 1.27 (0.422, 3.84) |

^a^ For definition of ischemic heart disease, see eTable S3

^b^ Not mutually exclusive. For instance a patient with a follow-up of 11 years would be included in both the >5-year- and the >10-year-categories.

^c^ Before or within 1 year after first renal biopsy indicating IgAN.

^d^ Before study entry (date of IgAN and corresponding date in reference individuals). For definitions, see eTable S1

^e^ Adjusted HR not possible to estimate due to lack of variation within strata.

^f^ CI:S not available, since the estimation of the variance-covariance matrix did not converge

py = person-years, HR = hazard ratio.

**eTable S7.** Risk of *ischemic heart disease (composite outcome)^a^* in patients with IgA nephropathy (IgAN) diagnosed in Sweden in 1974-2011, compared with *spouses* (corresponds to Fig. 2, column 3).

| **Variable** | **IgAN: Events/py** | **Ref: Events/py** | **Events/1000 py: IgAN vs. Ref** | **HR (95% CI)** |
| --- | --- | --- | --- | --- |
| Total | 290/35484 | 126/38893 | 8.2 vs. 3.2 | 1.91 (1.40, 2.61) |
| **Length of follow-up** | | | | |
| 0 to 1 years | 28/2344 | 6/2366 | 12 vs. 2.5 | 6.13 (1.15, 32.6) |
| 1 to 5 years | 58/11178 | 18/11492 | 5.2 vs. 1.6 | 1.73 (0.441, 6.75) |
| > 5 years^a^ | 204/34644 | 102/38293 | 5.9 vs. 2.7 | 1.81 (1.26, 2.6) |
| > 10 years^a^ | 138/30865 | 80/34816 | 4.5 vs. 2.3 | 1.93 (1.25, 3) |
| > 20 years^a^ | 33/17103 | 27/20806 | 1.9 vs. 1.3 | 3.57 (0.914, 13.9) |
| > 30 years^a^ | 9/5392 | 4/6344 | 1.7 vs. 0.63 | **e** |
| **Sex** | | | | |
| Women | 40/10970 | 55/10506 | 3.7 vs. 5.2 | 0.747 (0.373, 1.50) |
| Men | 250/24514 | 71/28387 | 10 vs. 2.5 | 4.49 (3.09, 6.53) |
| **Age** | | | | |
| ≤ 17 years | 2/1499 | 0/1530 | 1.3 vs. 0.0 | **e** |
| 18-39 years | 49/17405 | 24/17726 | 2.8 vs. 1.4 | 1.87 (0.83, 4.20) |
| 40-59 years | 157/13956 | 67/15863 | 11 vs. 4.2 | 1.64 (1.08, 2.48) |
| ≥ 60 years | 82/2624 | 35/3774 | 31 vs. 9.3 | 3.25 (1.52, 6.94) |
| **Calendar year of inclusion** | | | | |
| 1974-1988 | 68/8515 | 39/9516 | 8 vs. 4.1 | 1.9 (1.04, 3.47) |
| 1989-2001 | 167/17967 | 71/19859 | 9.3 vs. 3.6 | 1.75 (1.13, 2.71) |
| 2002-2015 | 55/9001 | 16/9518 | 6.1 vs. 1.7 | 2.20 (0.967, 4.99) |
| **Education** | | | | |
| Compulsory school (0-9 yrs) | 122/7285 | 46/8814 | 17 vs. 5.2 | 2.49 (1.25, 4.93) |
| Upper sec. school (1-3 yrs) | 110/15494 | 43/16648 | 7.1 vs. 2.6 | 2.89 (1.50, 5.58) |
| University | 49/12545 | 34/12930 | 3.9 vs. 2.6 | 0.687 (0.247, 1.91) |
| **Country of birth** | | | | |
| Nordic | 261/32593 | 119/35650 | 8.0 vs. 3.3 | 1.79 (1.29, 2.48) |
| Non-Nordic | 29/2891 | 7/3243 | 10 vs. 2.2 | 4.55 (0.774, 26.7) |
| **Henoch-Schönlein purpura (systemic IgA vasculitis)^c^** | | | | |
| Yes | 6/1271 | 3/1333 | 4.7 vs. 2.3 | **e** |
| **Cancer^d^** | | | | |
| No | 273/34006 | 121/37157 | 8.0 vs. 3.3 | 1.93 (1.39, 2.67) |
| Yes | 17/1478 | 5/1736 | 12 vs. 2.9 | **e** |
| **Diabetes^d^** | | | | |
| No | 269/34571 | 119/37711 | 7.8 vs. 3.2 | 1.97 (1.42, 2.72) |
| Yes | 21/913 | 7/1182 | 23 vs. 5.9 | **e** |
| **Other systemic inflammatory disease^d^** | | | | |
| No | 270/33347 | 114/36503 | 8.1 vs. 3.1 | 1.77 (1.28, 2.44) |
| Yes | 20/2137 | 12/2390 | 9.4 vs. 5.0 | **e** |
| **Cardiovascular disease (other than hypertension and IHD)^d^** | | | | |
| No | 231/31858 | 101/34371 | 7.3 vs. 2.9 | 1.85 (1.29, 2.64) |
| Yes | 59/3625 | 25/4522 | 16 vs. 5.5 | 2.18 (0.326, 14.5) |

^a^ For definition of ischemic heart disease, see eTable S3

^b^ Not mutually exclusive. For instance a patient with a follow-up of 11 years would be included in both the >5-year- and the >10-year-categories.

^c^ Before or within 1 year after first renal biopsy indicating IgAN.

^d^ Before study entry (date of first IgAN biopsy and corresponding date in reference individuals).

^e^ Adjusted HR not possible to estimate due to lack of variation within strata.

^f^ CI:S not available, since the estimation of the variance-covariance matrix did not converge

py = person-years, HR = hazard ratio

**Table S8.** Adjusted hazard ratios (HRs) for *acute myocardial infarction^a^* in patients with IgA nephropathy (IgAN) diagnosed in Sweden in 1974-2011, with the *general reference population.*

| **Variable** | **IgAN: Events/py** | **Ref: Events/py** | **Events/1000 py: IgAN vs. Ref** | **HR (95% CI)** |
| --- | --- | --- | --- | --- |
| Total | 216/56865 | 662/291433 | 3.8 vs. 2.3 | 1.83 (1.54, 2.18) |
| **Length of follow-up** | | | | |
| 0 to 1 years | 18/3904 | 26/19177 | 4.6 vs. 1.4 | 3.45 (1.61, 7.41) |
| 1 to 5 years | 51/18690 | 145/92314 | 2.7 vs. 1.6 | 1.50 (1.04, 2.15) |
| > 5 years^a^ | 147/55358 | 491/284850 | 2.7 vs. 1.7 | 1.89 (1.53, 2.34) |
| > 10 years^a^ | 99/48173 | 355/251930 | 2.1 vs. 1.4 | 1.81 (1.40, 2.33) |
| > 20 years^a^ | 25/25996 | 111/142430 | 0.96 vs. 0.78 | 1.52 (0.919, 2.52) |
| > 30 years^a^ | 5/7520 | 13/41811 | 0.66 vs. 0.31 | 2.62 (0.661, 10.3) |
| **Sex** | | | | |
| Women | 33/17434 | 106/88140 | 1.9 vs. 1.2 | 1.91 (1.22, 3.01) |
| Men | 183/39431 | 556/203293 | 4.6 vs. 2.7 | 1.82 (1.50, 2.21) |
| **Age** | | | | |
| ≤ 17 years | 1/5915 | 3/29913 | 0.17 vs. 0.10 | 5.78 (0.252, 132) |
| 18-39 years | 33/28397 | 90/141856 | 1.2 vs. 0.63 | 1.77 (1.16, 2.72) |
| 40-59 years | 106/18662 | 332/97644 | 5.7 vs. 3.4 | 1.82 (1.42, 2.34) |
| ≥ 60 years | 76/3891 | 237/22020 | 20 vs. 11 | 1.88 (1.38, 2.56) |
| **Calendar year of inclusion** | | | | |
| 1974-1988 | 43/11739 | 177/60986 | 3.7 vs. 2.9 | 1.58 (1.06, 2.37) |
| 1989-2001 | 126/28201 | 344/146094 | 4.5 vs. 2.3 | 2.13 (1.68, 2.69) |
| 2002-2015 | 47/16925 | 141/84353 | 2.8 vs. 1.7 | 1.54 (1.07, 2.21) |
| **Education** | | | | |
| Compulsory school (0-9 yrs) | 95/11173 | 237/60332 | 8.5 vs. 3.9 | 2.17 (1.58, 2.99) |
| Upper sec. school (1-3 yrs) | 83/26293 | 239/132747 | 3.2 vs. 1.8 | 1.82 (1.32, 2.53) |
| University | 31/18782 | 144/93079 | 1.6 vs. 1.5 | 1.22 (0.712, 2.09) |
| **Country of birth** | | | | |
| Nordic | 202/52692 | 617/269631 | 3.8 vs. 2.3 | 1.83 (1.52, 2.19) |
| Non-Nordic | 14/4167 | 45/21698 | 3.4 vs. 2.1 | 1.85 (0.909, 3.75) |
| **Henoch-Schönlein purpura (systemic IgA vasculitis)^c^** | | | | |
| Yes | 7/2704 | 17/13508 | 2.6 vs. 1.3 | 3.09 (0.862, 11.1) |
| **Cancer^d^** | | | | |
| No | 202/54387 | 616/278510 | 3.7 vs. 2.2 | 1.85 (1.54, 2.22) |
| Yes | 14/2478 | 46/12923 | 5.7 vs. 3.6 | 1.82 (0.445, 7.46) |
| **Diabetes^d^** | | | | |
| No | 192/55357 | 622/282514 | 3.5 vs. 2.2 | 1.81 (1.51, 2.17) |
| Yes | 24/1508 | 40/8919 | 16 vs. 4.5 | 1.09 (0.318, 3.70) |
| **Other systemic inflammatory disease^d^** | | | | |
| No | 196/53499 | 596/273055 | 3.7 vs. 2.2 | 1.88 (1.57, 2.26) |
| Yes | 20/3366 | 66/18378 | 5.9 vs. 3.6 | 0.793 (0.170, 3.71) |
| **Cardiovascular disease (other than hypertension and IHD)^d^** | | | | |
| No | 166/51072 | 510/259681 | 3.3 vs. 2.0 | 1.83 (1.50, 2.23) |
| Yes | 50/5793 | 152/31752 | 8.6 vs. 4.8 | 1.23 (0.707, 2.15) |
| **First-degree relative with IgAN** | | | | |
| Yes | 3/495 | 6/2580 | 6.1 vs. 2.3 | 2.52 (0.247, 25.7) |

^a^ For definition of myocardial infarction, see eTable S3

^b^ Not mutually exclusive. For instance a patient with a follow-up of 11 years would be included in both the >5-year- and the >10-year-categories.

^c^ Before or within 1 year after first renal biopsy indicating IgAN.

^d^ Before study entry (date of first IgAN biopsy and corresponding date in reference individuals).

^e^ Adjusted HR not possible to estimate due to lack of variation within strata.

py = person-years, HR = hazard ratio

# STROBE Statement—Checklist of items:

|  | Item No | Recommendation |
| --- | --- | --- |
| **Title and abstract** | 1✔ | (*a*) Indicate the study’s design with a commonly used term in the title **[p. 1]** |
|  |  | (*b*) Provide in the abstract an informative and balanced summary of what was done and what was found **[p. 5]** |
| Introduction | | |
| Background/rationale | 2✔ | Explain the scientific background and rationale for the investigation being reported **[p. 6]** |
| Objectives | 3✔ | State specific objectives, including any prespecified hypotheses **[p. 6]**  ***Comment***: We have expressed our hypothesis (and thereby our objective) in the last paragraph of our introduction. |
| Methods | | |
| Study design | 4✔ | Present key elements of study design early in the paper **[p. 6]** |
| Setting | 5✔ | Describe the setting, locations, and relevant dates, including periods of recruitment, exposure, follow-up, and data collection **[p. 6-7]** |
| Participants | 6✔ | (*a*) Give the eligibility criteria, and the sources and methods of selection of participants. Describe methods of follow-up **[p. 7-8]** |
|  |  | (*b*) For matched studies, give matching criteria and number of exposed and unexposed **[p. 7-8]** |
| Variables | 7✔ | Clearly define all outcomes, exposures, predictors, potential confounders, and effect modifiers. Give diagnostic criteria, if applicable **[p. 7-8]** |
| Data sources/ measurement | ✔8* | For each variable of interest, give sources of data and details of methods of assessment (measurement). Describe comparability of assessment methods if there is more than one group **[p. 7-8 + Supplementary tables]** |
| Bias | 9✔ | Describe any efforts to address potential sources of bias **[p. 15]** |
| Study size | 10✔ | Explain how the study size was arrived at  ***Comment***: This is a nationwide observational study in which we included all available patients with a biopsy report of IgA nephropathy. We did not perform any *a priori* power analysis; however, if the editor feels one is needed, we can carry out a post-hoc power analysis. |
| Quantitative variables | 11✔ | Explain how quantitative variables were handled in the analyses. If applicable, describe which groupings were chosen and why **[p. 8]** |
| Statistical methods | 12✔ | (*a*) Describe all statistical methods, including those used to control for confounding **[p. 9-10]** |
|  |  | (*b*) Describe any methods used to examine subgroups and interactions **[p. 9-10]** |
|  |  | (*c*) Explain how missing data were addressed **n/a** |
|  |  | (*d*) If applicable, explain how loss to follow-up was addressed **n/a** |
|  |  | (*e*) Describe any sensitivity analyses **[p. 10]** |
| Results | | |
| Participants | ✔13* | (a) Report numbers of individuals at each stage of study—e.g., numbers potentially eligible, examined for eligibility, confirmed eligible, included in the study, completing follow-up, and analysed **[p. 11]** |
|  |  | (b) Give reasons for non-participation at each stage  ***Comment***: Because this was a strict registry-based study, study participants were not contacted (all data were analyzed without knowledge of the identity of the study participants). Hence, we had no “non-participation” at different stages of the study. |
|  |  | (c) Consider use of a flow diagram  **Comment:** Dropouts occurred because of largely one reason, which is described in the text. |
| Descriptive data | ✔14* | (a) Give characteristics of study participants (e.g., demographic, clinical, social) and information on exposures and potential confounders **[p. 11, + table 1]** |
|  |  | (b) Indicate number of participants with missing data for each variable of interest **[table 1]** |
|  |  | (c) Summarize follow-up time (e.g., average and total amount) **[p. 11, 13 + table 1]** |
| Outcome data | ✔15* | Report numbers of outcome events or summary measures over time **[p. 11-13 + supplementary tables S5-S8]** |
| Main results | 16✔ | (*a*) Give unadjusted estimates and, if applicable, confounder-adjusted estimates and their precision (e.g., 95% confidence interval). Make clear which confounders were adjusted for and why they were included **[p. 8, p. 11-13 + supplementary table S5-S9]** |
|  |  | (*b*) Report category boundaries when continuous variables were categorized **table 1, supplementary tables S5-S8** |
|  |  | (*c*) If relevant, consider translating estimates of relative risk into absolute risk for a meaningful period **p. 11-13** |
| Other analyses | 17✔ | Report other analyses done—e.g., analyses of subgroups and interactions, and sensitivity analyses **[p.14-15 + Table 3 & 4 + Suppl. Table 1]** |
| Discussion | | |
| Key results | 18✔ | Summarize key results with reference to study objectives **[p. 13]** |
| Limitations | 19✔ | Discuss limitations of the study considering sources of potential bias or imprecision. Discuss both direction and magnitude of any potential bias **[p. 15]** |
| Interpretation | 20✔ | Give a cautious overall interpretation of results considering objectives, limitations, multiplicity of analyses, results from similar studies, and other relevant evidence **[p. 14-15]** |
| Generalizability | 21✔ | Discuss the generalizability (external validity) of the study results **[p. 14-15]** |
| Other information | | |
| Funding | 22✔ | Give the source of funding and the role of the funders for the present study and, if applicable, for the original study on which the present article is based **[p. 2]** |

*Give information separately for exposed and unexposed groups.

**Note:** An Explanation and Elaboration article discusses each checklist item and gives methodological background and published examples of transparent reporting. The STROBE checklist is best used in conjunction with this article (freely available on the Web sites of PLoS Medicine at http://www.plosmedicine.org/, Annals of Internal Medicine at http://www.annals.org/, and Epidemiology at http://www.epidem.com/). Information on the STROBE Initiative is available at http://www.strobe-statement.org.
